# Pericytes for Therapeutic Approaches to Ischemic Stroke

**DOI:** 10.3389/fnins.2021.629297

**Published:** 2021-06-18

**Authors:** Lu Cao, Yanbo Zhou, Mengguang Chen, Li Li, Wei Zhang

**Affiliations:** ^1^Department of Anesthesiology, Pain and Perioperative Medicine, The First Affiliated Hospital of Zhengzhou University, Zhengzhou, China; ^2^Department of Anesthesiology, Beijing Friendship Hospital, Capital Medical University, Beijing, China

**Keywords:** pericytes, ischemic stroke, cerebral blood flow, blood–brain barrier, therapeutic strategy

## Abstract

Pericytes are perivascular multipotent cells located on capillaries. Although pericytes are discovered in the nineteenth century, recent studies have found that pericytes play an important role in maintaining the blood—brain barrier (BBB) and regulating the neurovascular system. In the neurovascular unit, pericytes perform their functions by coordinating the crosstalk between endothelial, glial, and neuronal cells. Dysfunction of pericytes can lead to a variety of diseases, including stroke and other neurological disorders. Recent studies have suggested that pericytes can serve as a therapeutic target in ischemic stroke. In this review, we first summarize the biology and functions of pericytes in the central nervous system. Then, we focus on the role of dysfunctional pericytes in the pathogenesis of ischemic stroke. Finally, we discuss new therapies for ischemic stroke based on targeting pericytes.

## Introduction

Pericytes are perivascular multipotent cells located on capillaries. The perivascular cell was first discovered by Rouget in 1873, so it was named “Rouget’s cell” (Rouget, 1873). Furthermore, according to the morphology and location of Rouget’s cell, interacting with the underlying endothelial cells (ECs) that shared the basement membrane, Zimmermann renamed it “pericyte” in 1923 (Zimmermann, 1923). Subsequently, more studies discovered the origin and functions of pericytes. Pericytes in cerebral vessels develop from different layers of the germ. Faal et al. (2019) pointed out that pericytes of the central nervous system (CNS) were mainly derived from mesenchymal stem cells of the mesoderm. Some studies also discovered that pericytes of the forebrain originated from nerve crest cells, while pericytes of the midbrain, brain stem, and spinal cord originated from mesoderm stem cells (Etchevers et al., 2001; Korn et al., 2002; Kurz, 2009). There are pieces of evidence suggesting that pericytes in the brain can exert a large variety of functions (Bell et al., 2010; Armulik et al., 2011), including regulating cerebral blood flow (CBF), maintaining BBB integrity, regulating angiogenesis and inflammation, and acting as stem cells and progenitor cells (Gautam and Yao, 2018).

Ischemic stroke is a cerebrovascular disease due to the blockage of blood vessels and decreased blood supply in relevant brain regions (Li et al., 2014). When ischemic stroke occurs, a series of molecular and cellular events occur, ultimately leading to disruption of CBF, destruction of the blood—brain barrier (BBB), inflammation, glial cell activation, vascular malformation, and neuronal death (Moskowitz et al., 2010; Terasaki et al., 2014). Recent studies show that pericytes influence ischemic stroke pathology and contribute to progression and recovery.

In this review, we focus on recent studies on the biology and functions of pericytes during ischemic stroke. Specifically, we discuss the role of pericytes in CBF, BBB integrity, angiogenesis, immune response, and scar formation and fibrosis (Table 1). Finally, we address recent findings on treatment strategies for ischemic stroke by targeting pericytes.

**TABLE 1:** Functions of pericytes in ischemic stroke.

Functions	Roles	References
Regulating cerebral blood flow	Capillary contraction	Yemisci et al., 2009; Hall et al., 2014; Neuhaus et al., 2017; Kisler et al., 2020
Formation and maintenance of BBB	Regulation of vascular permeability Modulation of BBB integrity	Dohgu et al., 2005; Bai et al., 2015; Daneman and Prat, 2015
Angiogenic property	Blood vessel stabilization Revascularization	Dore-Duffy and LaManna, 2007; Stenzel et al., 2009
Immunological characteristics	Releasing anti-inflammatory cytokines/chemokines	Dohgu and Banks, 2013; Sakuma et al., 2016; Duan et al., 2018
Scar formation and fibrosis	Stem cell potential expressing PDGFRβ Neuroprotection	Makihara et al., 2015; Sakuma et al., 2016; Ozen et al., 2018

*BBB, blood—brain barrier; PDGFRβ, platelet-derived growth factor receptor-beta.*

### Pericytes and Cerebral Blood Flow in Ischemic Stroke

The question of whether pericytes can regulate CBF is still debatable. Pericytes have been demonstrated to regulate blood flow to the brain. For instance, actin and myosin-like filaments have been found in rat pericerebral cells (Le Beux and Willemot, 1978). Moreover, many kinds of contractile proteins, such as smooth muscle actin (SMA), have been identified in the culture of pericerebral cells, indicating that pericytes can synthesize the protein (Sieczkiewicz and Herman, 2003; Dore-Duffy and LaManna, 2007). Consistent with these findings, pericytes cultured *in vitro* have shown contraction due to intracellular Ca^2+^ (Kamouchi et al., 2004). Some studies reported that glutamate-induced capillary pericytes are mediated by prostaglandin E2 and nitric oxide, and vasodilation occurs before the arteriole dilation caused by electrical stimulation to increase blood flow (Hall et al., 2014; Kisler et al., 2017). Besides, Kisler et al. used pericyte-specific Cre mouse model with Cre-dependent human diphtheria toxin (DT) receptor (Kisler et al., 2020). DT led to rapid progressive loss of pericyte coverage of cortical capillaries; however, endothelial response, microvascular density, and neuron-evoked membrane potential responses remained. This study suggested that neurovascular uncoupling is driven by pericyte loss, not other vascular or neuronal dysfunction. The result supported the role of pericytes in CBF regulation (Kisler et al., 2020). Together, these results suggest that pericytes take an active part in regulating CBF under physiological conditions.

Pericyte contraction can be observed under pathological conditions as well, such as stroke. Research showed that in the process of stroke, pericytes could entrap red blood cells in the capillary contraction part, which obstruct the microcirculation (Yemisci et al., 2009). Similarly, Hall et al. (2014) reported that pericytes contracted capillaries and died quickly after ischemia, and pericyte death led to permanent capillary contraction; furthermore, the decreased time of CBF was prolonged even when arterial blood flow was restored. These pathological changes in pericytes after stroke were duplicated with the iCelligence electrical impedance system. Neuhaus et al. (2017) confirmed that chemical ischemia induced long-term and irreversible contraction of pericytes before death *in vitro*. These results suggest that the contraction and death of pericytes are involved in the pathogenesis of stroke by regulating CBF. Further studies have shown that the cause of pericyte death is partially mediated by glutamate, while free radical scavenging does not reduce pericyte death (Hall et al., 2014). Compared to this report, inhibition of nitrate-mediated oxidative stress has been demonstrated to reduce pericyte contraction induced by ischemia reperfusion and have a positive effect on tissue survival (Yemisci et al., 2009). In addition, in the rat model of focal cerebral ischemia, eddaravan, a free radical scavenger, can reduce infarct size by preventing pericyte contraction while promoting pericyte proliferation (Deguchi et al., 2014). Recently, Nelson et al. (2020) examined pericyte contractility using a new optogenetic model by inducing pericyte-specific CreER mouse line and ChR2 mouse and monitored and confirmed pericyte contractility *in vivo* and regulated capillary blood flow in the brain of aging mice under a two-photon microscope. Besides, Hartmann et al. (2021) also found that brain capillary pericyte optogenetic stimulation decreased lumen diameter and blood flow, but with slower kinetics than similar stimulation of mural cells on upstream pial and precapillary arterioles.

On the contrary, there are some researches indicating that pericytes cannot contract and regulate CBF as well. Using a variety of transgenic mice and two-photon microscopy, Hill et al. (2015) did not detect SMA expression in mice and human pericytes. They also showed that changes in blood vessel diameter and blood flow caused by optogenetic whisker stimulation and cortical spreading depolarization occurred in the microvessels covered by smooth muscle cell (SMC) but not in the capillaries covered by pericytes (Hill et al., 2015). In addition, by using a short-term middle cerebral artery occlusion (MCAO) model, it has been found that SMC contraction rather than pericyte contraction resulted in hypoperfusion and thus distal microvascular occlusion. Fernández-Klett et al. (2010) also found that the increase in CBF caused by neural activity can be explained by precapillary and penetrating arterioles, rather than pericyte in capillaries. These studies suggest that SMC, rather than pericyte, contributed to the regulation of blood flow under both physiological and pathological conditions. The difference is probably due to the similar structural and functional properties of pericytes and precapillary SMC (Hartmann et al., 2015), making it difficult to distinguish them completely. Some groups showed synaptic activity generated a synchronous Ca2^+^ drop in pericytes and SMCs resulting in telangiectasia starting mainly from primary or secondary capillaries and then spreading along arterioles and downstream capillaries, which may be the location of pericytes in the proximal capillaries as the main regulator of CBF (Cai et al., 2018; Khennouf et al., 2018; Rungta et al., 2018). However, the main divergence of the two opinions is the definition of pericyte, since Hill et al. (2015) and Fernández-Klett et al. (2010) defined SMA-expression capillary cell as SMC, while Hartmann et al., 2015 defined it as pericyte. The findings of Hill et al. (2015) and Fernández-Klett et al. (2010) are identical to other findings once definition differences are taken into account. In summary, most of the studies show that pericytes with SMA expression can regulate CBF.

### Pericytes and the Blood—Brain Barrier in Ischemic Stroke

**FIGURE 1:**
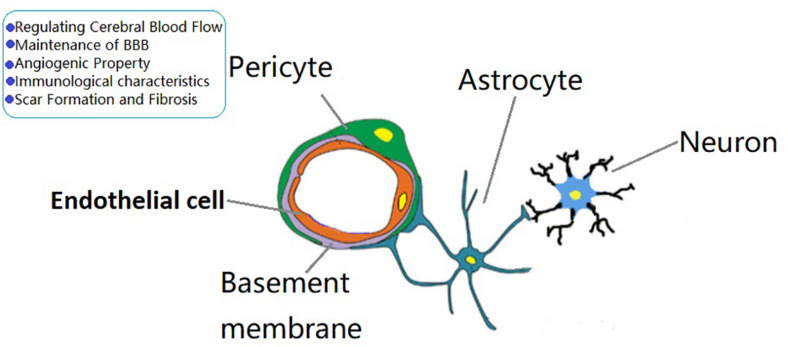
The role of brain pericytes at the neurovascular unit (NVU). A simplified NVU is composed of vascular cells [pericytes, vascular smooth muscle cells, endothelial cells (ECs)], glial cells (astrocytes, microglia, oligodendrocytes), and neurons. Pericytes have the functions of regulating cerebral blood flow, maintenance of blood—brain barrier permeability, angiogenic characteristcs, immune responses, and scar formation and fibrosis.

The BBB is a dynamic barrier between blood and brain tissue that acts as a selective barrier to substances. Pericytes play an important role in the formation and maintenance of the BBB. In the CNS, a simplified neurovascular unit is composed of vascular cells (pericytes, vascular SMCs, ECs), glial cells (astrocytes, microglia, oligodendrocytes), and neurons (Figure 1). Pericytes are important components of the neurovascular unit and BBB. Daneman et al. (2010) have found that pericyte recruitment occurred 1 week before astrocyte recruitment during the formation of BBB. They also found that the coverage of pericyte played a crucial role in vascular permeability, and pericytes regulated BBB at the endothelial connectivity level (Daneman and Prat, 2015). Bell et al. (2012) found that apolipoprotein E maintained cerebrovascular integrity necessary for normal neuronal function by regulating the cyclophilin A–nuclear-factor-κB–matrix metalloproteinase pathway in pericytes in an isoform-specific manner. Al Ahmad et al. (2009) found that the increased integrity of endothelial barrier was related to pericytes and astrocytes, and pericytes protected the endothelial barrier better than astrocytes after thymic ischemia.

Several studies have shown that the loss of pericyte coverage led to destruction of BBB and endothelial barrier functions. Pericytes maintain BBB function by releasing angiopoetin-1 (Ang-1) and transforming growth factor-beta1 (TGF-β1). Increased occludin expression in ECs is associated with pericyte release of Ang-1. However, low levels of occludin cause the breakdown of tight junction (TJ) and blood vessel permeability (Hori et al., 2004). Dohgu et al. (2005) discovered that pericyte-derived TGF-β1 had the ability to directly influence BBB function. TGF-β1 activates mitogen-activated protein kinase (MAPK) signaling to increase the expression of TJ protein and P-glycoprotein (P-gp) expressed in EC to improve BBB function. However, Shen et al. (2011) showed that TGF-β1 promoted tyrosine phosphorylation of vascular endothelial-cadherin and claudin-5 in ECs and enhanced paracellular permeability. The role of pericyte-derived TGF-β1 in ischemic stroke needs further studies. Besides, Zlokovic (2013) showed that loss of pericytes disrupted cerebrovascular integrity and led to microvascular reductions amplifying vascular damage.

Although pericyte dysfunction is not directly associated with ischemic stroke, pericytes may be indirectly involved in ischemic pathogenesis by affecting vascular integrity. There is evidence that pericytes regulate BBB integrity through vascular endothelial growth factor (VEGF), thereby regulating ischemic injury. Sodium cyanide (NaCN) was used in an *in vitro* ischemia model, and it has been found to significantly increase the expression of VEGF in brain pericytes, while the conditioned medium from NaCN-treated pericytes destroyed vascular integrity in the *in vitro* BBB model (Bai et al., 2015). Meanwhile, it has been shown that VEGF mediated the increased permeability of BBB in ischemic brain (Zhang et al., 2000). In contrast, prolonged exposure to VEGF has been shown to improve the integrity of the BBB after ischemia (Zechariah et al., 2013). This difference may be due to differences in dose, treatment measures, and timing. Furthermore, pericytes influence BBB integrity through reactive oxygen species (ROS), thereby affecting the ischemic process. Studies have shown that nicotinamide adenine dinucleotide phosphate (NADPH) oxidase 4 (NOX4) is highly upregulated in the peri-infarct area in the MCAO model. NOX4 has been shown to be primarily derived from pericytes, and the expression of NOX4 in pericyte was significantly increased in peri-infarct areas after MCAO. Further studies showed that the overexpression of NOX4 in pericyte caused BBB destruction through upregulation of metalloproteinase 9, especially emphasizing the significant role of ROS in BBB integrity (Nishimura et al., 2016).

### Angiogenic Property of Pericytes in Ischemic Stroke

Pericytes are closely related to the formation and stabilization of angiogenesis. The recruited pericytes enter new blood vessels to promote vascular maturation by releasing paracrine factors such as platelet-derived growth factor receptor-beta (PDGFRβ) (Dore-Duffy and LaManna, 2007). It has been reported that pericytes could stabilize the formation of capillary-like structures (Ramsauer et al., 2002). Persidsky et al. (2006) showed that pericytes were involved in the regulation of EC migration, proliferation, and differentiation. Furthermore, pericytes were reported to mediate the formation of new blood vessels through certain signaling molecules such as PDGFRβ, TGF-β, VEGF, and Ang-1 (Dore-Duffy and LaManna, 2007). Pericytes release VEGF to promote EC maturation (Hagedorn et al., 2004). Pericyte-derived TGF-β induces perivascular mesenchymal cells to differentiate into pericyte and SMCs (Sinha et al., 2004). Pericyte-released Ang-1 binds to Tie-2 in ECs, thereby promoting heparin binding to epidermal growth factor-like growth factor (HB-EGF) expression, which plays a key role in vascular stability (Stenzel et al., 2009).

Stroke is a cerebral blood circulation disease caused by stenosis, occlusion, or rupture of intracerebral arteries. Rebuilding blood flow to the brain in damaged areas aids in stroke recovery. Based on the important role of pericytes in angiogenesis, it is supposed that pericytes may contribute to stroke recovery by regulating angiogenesis. Recombinant human VEGF in the MCAO model has been shown to promote capillary growth and pericyte coverage, promote cerebral blood circulation, and thus reduce cerebral infarction area (Zechariah et al., 2013). In keeping with these observations, VEGF receptor inhibition accelerates cell death, inhibits EC proliferation, and exacerbates injury in neonatal stroke models (Shimotake et al., 2010). Based on these studies, it is speculated that pericytes have a positive effect in ischemic stroke by promoting angiogenesis.

It is important to point that angiogenesis may also have a negative effect on ischemic stroke. For example, elevated VEGF expression during ischemia was related to uncoupling of EC connectivity and increased vascular permeability and edema (Weis and Cheresh, 2005). In keeping with this finding, VEGF antagonism inhibited the formation of edema and tissue damage to some extent after MCAO in mouse brain (Nicholas van Bruggen et al., 1999). Furthermore, enhanced angiogenesis, including promoted EC activation and retinal hypervascularization, was observed in Notch1^±^ Notch3^–/–^ mice, a model of cerebral autosomal dominant arteriopathy with subcortical infarcts and leukoencephalopathy (CADASIL) (Kofler et al., 2015). In addition, an increased incidence of ischemic stroke was related to type 1 and type 2 diabetes (Janghorbani et al., 2007). Angiogenesis in diabetes is persistent and uncontrolled (Cheng and Ma, 2015). In a word, pericyte angiogenic properties may play a dual role in ischemic stroke due to differences in the animal model, timing, and types of injury.

### Immunological Characteristics of Pericytes in Ischemic Stroke

Pericytes take part in CNS defense by exhibiting non-specific and specific immune responses. A number of studies have reported that pericytes responded to pro-inflammatory signals. For example, chemokines and cytokines, including granulocyte colony-stimulating factor (G-CSF), interleukin (IL)-1 α, IL-6, and nitric oxide, are constitutively produced in brain pericytes of mice in normal conditions. In lipopolysaccharide (LPS)-induced inflammation, changes in expression levels of these chemokines and cytokines were accompanied by the induction of many new factors such as IL-5 and regulated upon activation normal T cell expressed and secreted factor (RANTES) (Kovac et al., 2011; Dohgu and Banks, 2013). With the increase of age and injury, pericytes with high expression of lysosomal acid phosphatase have phagocytosis. Pericytes may absorb substances in the blood or brain parenchyma through various endocytoses. When the BBB is broken down, pericytes can engulf the red blood cells (Castejon, 1984). Meanwhile, cultured rat pericytes expressed macrophage markers ED-2 and CD11b and were phagocytic with fluorescent dye-conjugated polystyrene beads and antibody-coated yeast polysaccharide, suggesting Fc receptor-independent and -dependent phagocytic activity (Balabanov et al., 1996). In summary, pericytes may have the immune cell-like characteristics and the ability to modulate immune responses.

Stroke is also a nervous system disease associated with the local inflammatory reactions and an immune response in the brain. It is supposed that pericytes may respond to CNS damage, including ischemic stroke, by transforming into microglia/macrophage-like cells (Boya, 1976). More recently, some studies have suggested that pericytes may acquire the microglia-like properties after ischemic stroke. First, it has been reported that the cells that express the regulator of G-protein signaling 5 (RGS5) after cerebral ischemia injury were mainly pericytes (Bondjers et al., 2003; Cho et al., 2003; Berger et al., 2005), which is associated with the proliferation and production of CD11b + and galactosin 3 + microglia cells (Ozen et al., 2018). Secondly, pericytes of the human brain acquire stemness characteristics and differentiate into a variety of lineages including microglia cells under oxygen and glucose deprivation simulating ischemic injury *in vitro* (Nakagomi et al., 2015). Meanwhile, pericyte stemness characteristics were detected in the ischemic regions of the mice, indicating that pericytes had multipotency in ischemic induction (Nakagomi et al., 2015; Gouveia et al., 2017). In addition, Iba1 + microglia cells express PDGFRβ in ischemic encephalopathy, showing that some microglia may come from post-ischemic pluripotent pericytes (Sakuma et al., 2016). Additionally, PDGFRβ + pericytes isolated from ischemic differentiated into microglia-like cells and acquired phagocytic activity (Sakuma et al., 2016). Recently, it has been reported that within 2 h of systemic inflammation, PDGFRβ mural cells of blood vessels rapidly secreted chemokine CC-chemokine ligand 2 (CCL2), which in turn increased total neuronal excitability by promoting excitatory synaptic transmission in glutamatergic neurons of multiple brain regions (Duan et al., 2018). The study demonstrated *in vivo* that PDGFRβ cells functioned as initial sensors of external insults by secreting CCL2, which relayed the signal to the CNS. Through their gateway position in the brain, PDGFRβ cells are ideally positioned to respond rapidly to environmental changes and to coordinate responses. In summary, these studies show that pericytes can differentiate into microglia-like cells and perform related functions in ischemic conditions.

### Pericytes and Scar Formation and Fibrosis in Ischemic Stroke

After injury of the CNS, glial cells are activated and form glial scar at the injury region by deposition of chondroitin sulfate proteoglycans, including neuroproteoglycans and phosphatase proteoglycans (Fawcett and Asher, 1999; McKeon et al., 1999; Silver and Miller, 2004; Li et al., 2013; Cregg et al., 2014). The function of scar tissue can keep toxic substances from spreading throughout the CNS (Fitch and Silver, 2008; Kawano et al., 2012). However, excessive or prolonged scar formation can inhibit axonal regeneration and hinder the recovery process, leading to fibrosis (Höke and Silver, 1996; Asher et al., 2001; Fitch and Silver, 2008). Besides, it is reported that astrocyte scar formation contributes to rather than prevents CNS axon regeneration (Anderson et al., 2016). The study showed that preventing or attenuating scar-forming astrocytes or ablating chronic astrocytic scars all failed to generate spontaneous regrowth of serotonergic axons in spinal cord injury (SCI) lesions. Sustained local delivery *via* hydrogel depots of required axon-specific growth factors not present in SCI lesions stimulated robust, laminin-dependent sensory axon regrowth past scar-forming astrocytes and inhibitory molecules in SCI lesions. By contrast, preventing astrocytic scar formation significantly reduced this stimulated axon regrowth.

It has been suggested that pericytes promoted scar formation and organ fibrosis. Pericytes are categorized into type I (Nestin-GFP**^–^**/NG2-DsRed**^+^**) and type II (Nestin-GFP**^+^**/NG2-DsRed**^+^**), producing adipocyte/fibroblast and nerve/myogen cells, respectively (Birbrair et al., 2013a,b). It has been reported that pericytes of type I gathered and contributed to scar formation in multiple organs (including brain, spinal cord, myocardium, and kidneys) after injury (Birbrair et al., 2014). It has been reported that in ischemic injury, PDGFRβ**^+^** pericytes induced the fibrosis response in the kidney and CNS (Chen et al., 2011; Makihara et al., 2015). Compared to the control group, PDGFR**^±^** mice showed reduced fibrosis, reduced fibronectin deposition, and increased infarct size in the ischemic area, showing that PDGFRβ signaling-induced fibronectin production was essential for the repair process after ischemic stroke (Makihara et al., 2015). This discovery agrees with the neuroprotective effect of fibronectin in CNS injury (Tom et al., 2004; Yanqing et al., 2006).

### Pericytes for Therapeutic Approaches to Ischemic Stroke

As discussed above, pericytes are involved in maintaining normal cerebrovascular function and play an important role in the pathological process of ischemic stroke. Thus, targeting pericytes may be an effective therapeutic method for ischemic stroke (Dalkara, 2019).

RGS5 protein that regulates vascular development was identified as a biomarker of pericytes (Bondjers et al., 2003). Ozen et al. (2018) found that the number of pericytes increased and the damage to the BBB decreased significantly after the RGS5 gene was knocked out in the model of permanent midbrain occlusion. In addition, loss of RGS5 in pericytes maintained aquaporin-4 (AQP4) expression in astrocytes and the integrity of TJs in ECs, reduced cerebral hypoxia, alleviated vascular leakage, and protected neurons in ischemic position (Ozen et al., 2018). AQP4 is a water channel protein expressed on astrocytic endfeet and plays an important role in BBB integrity. TJs have an important role in the maintenance of the BBB, and their unique expression in the brain correlates with BBB permeability. In acute stroke, there is degradation of TJs, resulting in loss of vascular integrity. Thus, targeting RGS5 might be a potential therapeutic strategy for ischemic stroke. Alarcon-Martinez et al. (2019) found that the α-SMA-mediated contractility in ischemic stroke and the effect of calcium in regulating contractile response could help in understanding the pericytes related to CBF at single-capillary level in ischemic stroke. Sun M. et al. (2020) found that sentrin/SUMO-specific protease 1 (SENP1) deletion in pericytes exacerbated infarct size and motor dysfunction after cerebral ischemia, though it had no effect on cognitive function. They also found that the deletion of pericyte-specific SENP1 significantly exaggerated neuronal damage after cerebral ischemia in mice. The knockdown of SENP1 in pericytes could activate apoptotic pathways and destroy the integrity of the cell barrier *in vitro*. These findings suggested that targeting SENP1 in pericytes might be a new therapeutic method for ischemic stroke (Sun M. et al., 2020). Besides, a study found that intracerebroventricular pleiotrophin (PTN) infusions prevented neuronal apoptosis in pericyte-ablated mice from persistent circulatory changes. PTN is a neurotrophic growth factor. The silencing of pericyte-derived PTN rendered neurons vulnerable to ischemic injury. This study demonstrated that pericyte loss was closely related to acute circulatory collapse and loss of PTN support. These findings suggested PTN support might be a new therapeutic method (Nikolakopoulou et al., 2019). Recently, Shibahara et al. (2020) demonstrated that pericyte-mediated fibrosis repair *via* PDGFR promotes functional recovery by enhancing peri-infarct oligodendrocyte formation and astrocyte proliferation following acute ischemic stroke. Besides, pericytes develop multipotency following experimental ischemia in mice, and these ischemia-induced multipotent stem cells (iSCs) can contribute to neurogenesis. This property of pericytes showed great potential in the treatment of neurovascular diseases (Geranmayeh et al., 2019). Pericytes from induced pluripotent stem cells (iPSC) are also expected to be used in autotransplantation therapy in ischemic stroke as acquiring BBB characteristics and binding to astrocytes, ECs, and neurons (Faal et al., 2019; Stebbins et al., 2019). Pericytes have been shown to acquire properties similar to stem cells and microglia after cerebral ischemia, providing another potential therapeutic strategy for recovery from ischemic stroke (Özen et al., 2014; Nakagomi et al., 2015). Recently, Sun J. et al. (2020) generated pericyte-like cells (PCs) from human pluripotent stem cells (hPSCs). They found the cranial neural crest-derived pericyte-like cells (hPSC-CNC PCs) expressed typical pericyte markers and showed distinct pericyte properties. Moreover, when transplanted into a mouse model of MCAO with BBB breakdown, hPSC-CNC PCs efficiently improved neurological functional recovery in MCAO mouse model. The study indicated that hPSC-CNC PCs might represent an ideal cell source for the treatment of BBB dysfunction-related disorders and might be a new therapeutic method for ischemic stroke (Sun J. et al., 2020).

Although significant progress has been made in understanding the function of pericytes in the pathogenesis of ischemic stroke, some key issues remain to be studied further. First, although various markers have been used to identify pericytes, there are currently no pericyte-specific markers. It is important to note that the pericytes in most studies contained PDGFR^+^ cells including both pericytes and SMCs. Secondly, pericytes are a diverse population of cells, and different types of pericytes may play different roles in ischemic stroke. The study in the future should focus on the identification of pericyte-specific and isotype-specific biomarkers, as well as drugs targeting pericytes or modulating their activities and autotransplantation of pericytes. Acquiring more information about pericytes will help us in studying pericytes in a novel way and facilitating the development of therapies for ischemic stroke.

## Author Contributions

LC searched for relevant literature and drafted the manuscript. YZ and MC searched for relevant literature and revised the manuscript critically. LL and WZ provided professional guidance for this review and performed a final check of the manuscript. All authors contributed to the review of this manuscript and approved the submitted version.

## Conflict of Interest

The authors declare that the research was conducted in the absence of any commercial or financial relationships that could be construed as a potential conflict of interest.
